# Knowledge and attitudes toward epilepsy among school teachers in West of Iran

**Published:** 2015-07-06

**Authors:** Narges Karimi, Mohammad Heidari

**Affiliations:** 1 Department of Neurology, School of Medicine, Mazandaran University of Medical Sciences, Sari, Iran; 2 Department of Epidemiology and Biostatistics, School of Health, Kerman University of Medical Sciences, Kerman, Iran

**Keywords:** Epilepsy, Attitude, Knowledge, Teachers, Students

## Abstract

**Background:** Epilepsy comprised the highest proportion of neurological problem of childhood stage, which observed mostly in the first decade of life. The dramatic effect of having a seizure in the classroom can be very traumatic for any child. The knowledge and attitude of teachers toward epilepsy have a direct impact on the life of students with epilepsy.

**Methods: **A cross-sectional descriptive survey was conducted in Kermanshah (West of Iran). 305 teachers from 25 public schools were randomly participated in this study. The questionnaire included 39 items and three sections (demographic information, knowledge, and attitude about epilepsy).

**Results: **In this study, 97% participants had heard or read about epilepsy. Attitude and knowledge about epilepsy was positive in weighted sum of the item responses, but there were deficits in individual items and first-aid management of seizure attacks. There was no meaningful relationship between attitude scores and demographic items, but higher level of education, female gender, and marital status had a positive influence on teachers’ knowledge toward children with epilepsy.

**Conclusion:** The main findings indicated a good knowledge and positive attitude about epilepsy among school’s teachers. Nevertheless, there is still a need to improve certain aspects of knowledge and attitude and first aid management of an epileptic attack among teachers.

## Introduction

Epilepsy is one of the most common neurological problems which affected about 1% of the world’s population.^1^ A prevalence rate of 0.7-1.8% has been reported in Iran.^2^ It is the one the most prevalent serious brain disorder of societies that involves people in the different age groups, races, and social classes.^3^^,^^4^ This disorder comprised the highest proportion of neurological problem of childhood stage, which observed mostly in the first decade of life.^5^ Globally, there are almost 33 million children with epilepsy, and it is estimated that they have 2-5 times more chances to present behavioral, emotional, and psychiatric problems in comparison with healthy children or children with other chronic diseases.^6^ In Iran, in every 1000 school-aged children, there are 4.2 youngsters suffering from epilepsy. Furthermore, 65% of those affected by epilepsy are teenagers and children.^7^ The dramatic effect of having a seizure in the classroom can be very traumatic for any child. The children suffering from epilepsy are often stigmatized because of fear of the seizure in public and loss of self-control.^8^^,^^9^

Children with epilepsy categorized as students who are at high risk for educational underachievement, learning disability, mental health problem, social isolation, and poor self-steam.^5^^,^^10^^,^^11^ The knowledge and attitude of teachers toward epilepsy have a direct impact on life of those students in several senses, such as: school performance, social skill development, and after school success in the areas of employment, social skills, and social network development.^12^ Teachers without experience of epilepsy often think that children with epilepsy are victims of bullying and their integration into the school collective is problematic.^13^ Several studies indicated that epilepsy has been associated with misconceptions and misbeliefs, which led to stigmatization and discrimination.^14^^,^^15^ This study aimed to evaluate the knowledge and attitude of teachers toward epilepsy in order to identify the needs and requirements of students with epilepsy in the schools.

## Materials and Methods

This cross-sectional study was conducted in surrounding of Kermanshah province, Iran, with a population of 1,945,227 people. This is considered as regional pole for education, which has a several public and private primary and secondary schools and four public universities. 305 teachers from 25 public schools were randomly invited to participate.

The questionnaire included 39 items that were developed after an extensive review of the international literature. These questions were divided into three sections: Demographic information and familiarity with epilepsy (12 items), attitude (15 items), and knowledge about epilepsy (12 items).

We first translated the questionnaire into Persian and then it was localized by consulting three experts in the field of neurology in Iran. A pilot study was conducted in order to test the questionnaire for reliability and validity. Validity of the instrument was tested by Cronbach’s alpha test which showed 0.7 consistency between questions. The reliability of questionnaire also was checked by test-retest among 20 teachers. There is no significant difference in teachers’ responses in two interviews. These teachers were excluded from final sample of the study. In the subscale of knowledge, 7 items assessed medical-related knowledge about epilepsy (e.g., causes, treatment, seizure triggers), and 5 items were related the social aspects of the disorder (e.g., the individual with epilepsy doesn’t possess a normal life expectancy). The attitude subscale consisted ten items assessed respondent’s feeling about being in social contact with epilepsy, five items were related to limitations and concealment of epilepsy. Responses to attitude and knowledge items assessed through 5-point Likert-type scale with following options: Strongly agree, agree, neither agree nor disagree, disagree, and strongly disagree.

In addition, familiarity with epilepsy included six items of certain questions i.e. Have you ever heard about epilepsy? Have you ever been student with epilepsy? Did you know the first-aid management of seizure? Have you seen an epileptic fit?

A questionnaire was administered on 305 school teachers from different parts of Kermanshah. Statistical software SPSS for Windows (version 13.0, SPSS Inc., Chicago, IL, USA), the independent sample t-tests and one-way ANOVA were used for analysis of data. Statistical significance considered as (P < 0.0500). The regression was performed to determine the effects of demographic and familiarity with epilepsy on knowledge and attitude scales.

## Results

***Demographics***

The sample included 218 women (71.5%) and 87 men (28.5%) aged 22-55 years (mean = 38.4 years, standard deviation = 6.2). Out of 305 teachers, 91.8% of the subjects were married and 8.2% were single. With respect to the level of education, 3.6% had completed high schools, 25.2% had associated diploma, 64.9% had bachelor, and 6.3% were holding master or higher degree. With affect to the years of teaching experiences, 2% taught 1-3 years, 4.9% 3-6 years, 8.5% 6-10 years, and 84.6% 10 years or higher.

***Familiarity with epilepsy***

Six questions were related to familiarity with epilepsy. The data related to results these questions are presented in table 1. 97% of respondents were heard about epilepsy. 29.8 reported that they have previously taught a student with epilepsy, but only 6.2% reported that were presently teaching a student with epilepsy. There was no significant relationship between these groups.

About 61.3% of teachers had observed an epileptic fit, but only 40% responders explained the first-aid management of seizure. All of them believed that putting an object in the mouth of the students during an epileptic seizure can prevent tongue injuries. This improper knowledge was associated with a higher education (P = 0.0200) and years of experience teaching (P = 0.0010). 82% of respondents knew symptoms of seizure. There was a significant relationship between understanding of seizure’s symptoms and females gender (P = 0.0300) and also years of teaching experience (P = 0.0400).

***Attitude toward Epilepsy***

Analysis of scores of attitude part of the questionnaire included evaluation of the weighted sum of the item responses and individual item analysis. Weighted sums of the item responses provide a quantity of the teacher total attitude, with higher scores demonstrating a more agreeable attitude. Scores for the 15 items were ranged from 15 to 75. Analysis of the responses of individual item was showed to calculate teachers’ scores on the item. Table 2 lists the attitude items and the mean responses of the participants in this study. To test the possible relationships between demographic variables and familiarity with epilepsy with attitude scale scores, a backward regression analysis was concluded. According to the statistical analysis, there was no significant association between attitude scores of the teachers and their gender, marital status, level of education, and years of teaching experience with epileptic of students.

***Knowledge about Epilepsy***

Knowledge scale includes 12 items. Weighted sums of the responses provide measures of the respondents’ knowledge about epilepsy with higher scores are demonstrating more good knowledge. Table 3 shows the individual knowledge items and the scores of the participants. To assess the relationships between teacher demographic characters (age, gender, marital, education level, and years of teaching) and familiarity with epilepsy with knowledge scores, a second regression analysis was conducted. The results of the regression analysis were summarized in the table 4. There was a significant association between knowledge score with female gender, marital status, and higher level of education. 

**Table 1:** Summarized results of questions regarding understanding of the epilepsy and demographic scores

**Result**	**Q1**	**Q2**		**Q3**		**Q4**		**Q5**		**Q6**	
	**Yes (%)**	**Yes (%)**	**No (%)**	**Yes (%)**	**No (%)**	**Yes (%)**	**No (%)**	**Yes (%)**	**No (%)**	**Yes (%)**	**No (%)**
Total	97.0	82.0	18.0	29.8	70.2	6.2	93.8	61.3	38.7	40.0	60.0
Gender											
Male	27.2	21.3	7.2	8.5	20.6	2.6	25.9	18.7	9.9	12.5	16.1
Female	69.8	60.7	10.8	21.3	49.6	3.6	67.9	42.6	28.8	27.5	43.9
Age											
22-33	14.8	11.8	3.6	3,9	11.5	0.7	14.8	9.2	6.2	5.9	9.5
34-44	66.2	56.8	11.1	21,9	46.8	4.9	63.0	43.0	24.9	27.9	40.0
45-55	16.1	13.4	3.3	4,9	11.8	0.7	16.1	9.2	7.5	6.2	10.6
Education level											
Diploma	3.3	2.6	1.0	0.3	3.3	0.3	3.3	1.6	2.0	0.3	3.3
Associated Diploma	24.3	19.0	6.2	6.2	19.0	1.3	23.9	13.8	11.5	8.5	16.7
Bachelor	63.3	55.7	9.2	21.3	43.3	4.3	60.7	41.3	23.6	28.5	36.4
Master and higher	6.2	4.6	1.6	2.0	3.9	0.3	5.9	4.6	1.6	2.6	3.6
Years of teaching experience											
1-3	2.0	1.0	1.0	0.3	1.8	0.3	1.6	1.0	1.0	0.7	1.5
4-6	4.6	4.3	0.7	2.0	3.6	0.3	4.6	3.0	2.0	2.6	2.5
7-10	8.5	6.6	2.0	3.3	5.5	0.7	7.9	4.9	3.6	3.6	4.9
˃ 10	81.6	70.2	14.1	24.3	59.3	4.9	79.7	52.4	31.8	33.1	51.1

Q1: Have you ever heard or read about epilepsy?; Q2: Did you know symptoms of epilepsy?; Q3: Have you ever been students with epilepsy in your classroom?; Q4: Did you were currently teaching a student with epilepsy?; Q5: Have you ever seen an epileptic fit?; Q6: Did you know how to manage the seizure for the first time?

**Table 2:** Attitude items and scores of participants

**Attitude item**	**Mean ± SD**
Schools should not place children with epilepsy in regular classrooms	2.58 **± **0.99*
Persons with epilepsy have the same rights as all people	4.28 **± **0.69
Persons with epilepsy can safely drive	2.11 **± **0.92*
The onset of an epileptic seizure in a spouse is sufficient reason for divorce	±0.81*
Children with epilepsy should attend regular public schools	** ± **0.92
Persons with epilepsy are a danger to the society	1.80 ± 0.74*
Individuals with epilepsy are accident-prone	3.77 ± 0.82
Children of school had to be kept away from classmates who have epilepsy	3.77 **± **0.86
Persons with epilepsy cannot marry with persons without	2.23 ± 0.81*
Epilepsy	3.73 ± 0.78
I allow any child to sit in the same class with a child with epilepsy	3.50 **± **0.93
Equal employment opportunities should be available to individuals with epilepsy	1.62 **± **0.85*
The cause of epilepsy is insanity	3.73 **± **0.84
I allow any child to play with a child with epilepsy	2.33 **± **0.88*
Epilepsy is a kind of incurable disorder	2.48 **± **0.86*
Children with epilepsy have a higher incidence of psychosis than normal children	

Note. Scales ranges from 1 (Strongly disagree) to 5 (Strongly agree); SD: Standard deviation;

* Items for which a “disagree” response (scored lower than 3) indicates a positive attitude

**Table 3:** Items of knowledge and responders scores

**Knowledge item**	**Mean ± SD**
The individual with epilepsy does not possess a normal life expectancy	2.45 ± 0.99*
Persons with epilepsy are mentally retarded	2.13 ± 0.80*
Persons with epilepsy can safely participate in strenuous activity	2.61 ± 0.96*
Epilepsy is a disorder of infections	1.63 ± 0.77*
Epilepsy is a disorder of the brain	3.87 ± 0.79
The offspring of parents with epilepsy will also have epilepsy	2.67 ± 0.94*
Persons of epilepsy should not drive	3.56 ± 0.98
Persons of epilepsy should not climb	3.37 ± 0.98
Inadequate steep can cause attacks of seizure in persons with epilepsy	3.66 ± 0.72
Hungry cause attack of seizure in persons with epilepsy	3.45 ± 0.76
Some certain foods or drinks make a seizure	3.60 ± 0.70
Persons with epilepsy need to use drug	3.77 ± 0.82

Note: Scales ranges from 1 (Strongly disagree) to 5 (Strongly agree); SD: Standard deviation;

* Item for “disagree” response (scored lower than 3) indicates a good knowledge

**Table 4:** Results of the regression analysis of variables on knowledge scale scores among teachers

**Predator**	**B**	**B**	**T**	**P**
Gender	0.091	0.121	2.015	0.0145
Marital status	-0.150	-0.124	-2.106	0.0360
Education	-0.036	-0.066	-1.103	0.0266
Years teaching	-0.018	-0.034	0.574	0.5670
Teach now	0.014	0.032	0.547	0.5840
Have taught	0.051	0.021	0.342	0.7320
Know epilepsy	0.051	0.058	0.908	0.3650
Epileptic fit	0.023	0.036	-0.550	0.5830
First aid	0.004	0.006	0.094	0.9250
Heard epilepsy	0.012	0.006	0.0102	0.9190

Note. Teach now: Currently teaching a student with epilepsy; Have taught: Have previously taught a student with epilepsy; Know epilepsy: Know the symptom of epilepsy; Seen epileptic fit: Has seen an epileptic fit; First aid: Knows the first aids in the management of epileptic fit; Heard epilepsy: Has ever heard or read about epilepsy

## Discussion

In this study, the most of teachers heard or read about epilepsy. Awareness about epilepsy is shown to be high in several studied.^16^^-^^21^ In contrast, the awareness about epilepsy among school teachers in Thailand was limited to 57.8%.^22^ The reason for this difference is not clear, but it may be due to close relationship population and public health education and also experience teaching a student with epilepsy. The present study showed female teachers and who had higher years of teaching, knew symptoms of epilepsy better than other teachers. It could be the result of higher level of communication between the teachers, especially female teachers, and epileptic students. In our study, participants have shown a positive attitude toward epilepsy similar to finding in other literatures.^4^^,^^5^^,^^9^^,^^16^^,^^23^ On the other hand, they had a better attitude than subjects in previous studies.^1^^,^^7^^,^^24^ This result is promising and encouraging. There was no significant relationship between attitude scale and demographic items and familiarity with epilepsy. Bishop showed that there was a relationship between higher levels of education and years of teaching experiences with higher scores in the attitude scale.^5^ In Turkish study found young teachers age and male gender predictive of positive attitude.^25^ However, the attitude score was positive, but individual item analysis showed different issues. For example 1/3 of respondents accepted as true that children with epilepsy have a higher incidence of psychosis in comparison to the normal children and also some teachers believed that epilepsy could be enough reason to prevent marriage or for divorce. Mustapha et al*.* Reported similar findings in their study among school teachers in Osogbo.^16^

This is believed that children with epilepsy should be separated from normal students and they are making distressing in social learning. 3.3% responders agreed that epilepsy is a form of in the insanity, which is more positive than the results of previous studies performed in Iran^1^ and other Asia countries.^4^^,^^12^^,^^18^^,^^20^^,^^21^ Furthermore, negative association between insanity and epilepsy has been reported by developed countries such as, USA, Denmark, and Italian.^26^^-^^28^ Relation of insanity to epilepsy has been considered true from ancient times despite of scientific evidence in the countries to reject this. Regarding treatment the most of teachers believed that epilepsy is a kind of curable disorder similar to results reported in the previous studies.^18^^,^^21^^,^^22^^,^^29^ This belief made the children refer to physicians and ability to present their selves in the society.

The teacher combination scores on the knowledge scale also showed a positive drift in all items. The most of the participants knew that hungry, some foods and also inadequate sleep may lead to seizure attacks. These results are very encouraging because having awareness about these topics caused that they cared more for nutrition of the children. In the study of Akpan, poor knowledge of seizure disorder with respect to the cause, diagnosis, and treatment is noted among school teachers.^30^ Regarding the knowledge about the cause of epilepsy, the most of teachers acknowledged that epilepsy is a disorder of the brain and persons with epilepsy need to use the drug for control their seizure. These acquaintances caused that teachers stimulated students for following-up of their treatment. There was a significant association between knowledge scores with female gender, marital status, and higher level of education. Ghanean et al. reported similar findings in their study among public in Tehran.^31^

In terms of the first time management, the majority of teachers were not familiarized with the initial procedures management of seizure attack. Contrary to standard first- aid management of epileptic fit,^32^ all of the teachers who answered this question would be somehow inappropriate, similar to other studies.^5^^,^^9^^,^^16^^,^^24^^,^^33^ Our study found a correlation between this misconception and higher level of education and teaching experience. This finding may be reflected to several reasons, containing attained from unreliable resources, and poor educative programs about epilepsy. This result suggested poor educative programs about epilepsy. This unfamiliarity can be a source of a serious problem for children with epilepsy when seizure fit occurred in schools.

Sufficient information about epilepsy caused that children were able to existing in society properly and lived likely other children. Increase awareness of teachers about epilepsy is necessary due to the role of teachers in the psychosocial development and quality of life of students with epilepsy. Mass media and physicians have an important role in the teachers’ knowledge about epilepsy.

***Limitation***

Present study was performed in an urban population. Therefore, these results cannot be generalized countrywide due to extensive cultural differences between urban and rural areas.

## Conclusion

This study showed overall good knowledge of epilepsy and positive attitudes toward epilepsy among school’s teachers. Although there were deficits in some of the items and first - aids management of seizure. In general, the participants with a higher level of education showed better answers about knowledge of epilepsy.

## Notes:

**How to cite this article: **Karimi N, Heidari M. Knowledge and attitudes toward epilepsy among school teachers in West of Iran. Iran J Neurol 2015; 14(3): 130-5.

## References

[1] Asadi-Pooya Aa, Torabi-Nami M (2012). Knowledge and Attitude Towards Epilepsy Among Biology Teachers in Fars Province, Iran. Iran J Child Neurology.

[2] Maryam S, Parviz B (2013). Depression in children and adolescents with epilepsy: a 15 year research review of prevalence, and demographic and seizure related correlates. Iran J Pediatr.

[3] Jacoby A (2002). Stigma, epilepsy, and quality of life. Epilepsy Behav.

[4] Aydemir N (2008). Developing two different measures for assessing knowledge of and attitudes toward epilepsy for the Turkish population. Epilepsy Behav.

[5] Bishop M, Boag EM (2006). Teachers' knowledge about epilepsy and attitudes toward students with epilepsy: results of a national survey. Epilepsy Behav.

[6] Toli T, Sourtzi P, Tsoumakas K, Kalokerinou-Anagnostopoulou A (2013). Association between knowledge and attitudes of educators towards epilepsy and the risk of accidents in Greek schools. Epilepsy Behav.

[7] Reyace H, Kaheni S, Sharifzadeh G (2014). Teachers' knowledge about epilepsy. J Mazandaran Univ Med Sci.

[8] Dantas FG, Cariri GA, Cariri GA, Ribeiro Filho AR (2001). Knowledge and attitudes toward epilepsy among primary, secondary and tertiary level teachers. Arq Neuropsiquiatr.

[9] Alkhamra H, Tannous A, Hadidi M, Alkhateeb J (2012). Knowledge and attitudes toward epilepsy among school teachers and counselors in Jordan. Epilepsy Behav.

[10] Williams J (2003). Learning and behavior in children with epilepsy. Epilepsy Behav.

[11] Austin J (2000). Impact of epilepsy in children. Epilepsy Behav.

[12] Hsieh LP, Chiou HH (2001). Comparison of epilepsy and asthma perception among preschool teachers in Taiwan. Epilepsia.

[13] Brabcova D, Lovasova V, Kohout J, Zarubova J (2012). Familiarity with and attitudes towards epilepsy among teachers at Czech elementary schools--the effect of personal experience and subspecialization. Seizure.

[14] DiIorio C, Osborne SP, Letz R, Henry T, Schomer DL, Yeager K (2003). The association of stigma with self-management and perceptions of health care among adults with epilepsy. Epilepsy Behav.

[15] Ablon J (2002). The nature of stigma and medical conditions. Epilepsy Behav.

[16] Mustapha AF, Odu OO, Akande O (2013). Knowledge, attitudes and perceptions of epilepsy among secondary school teachers in Osogbo South-West Nigeria: a community based study. Niger J Clin Pract.

[17] Gzirishvili N, Kasradze S, Lomidze G, Okujava N, Toidze O, de Boer HM (2013). Knowledge, attitudes, and stigma towards epilepsy in different walks of life: a study in Georgia. Epilepsy Behav.

[18] Thacker AK, Verma AM, Ji R, Thacker P, Mishra P (2008). Knowledge awareness and attitude about epilepsy among schoolteachers in India. Seizure.

[19] McLin WM, de Boer HM (1995). Public perceptions about epilepsy. Epilepsia.

[20] Homi BN, Rehman A, Saleh S, I, Zehra N (2014). Knowledge, attitude and practices of school teachers towards epileptic school children in Karachi, Pakistan. Pak J Med Sci.

[21] Choi-Kwon S, Park KA, Lee HJ, Park MS, Lee CH, Cheon SE (2004). Familiarity with, knowledge of, and attitudes toward epilepsy in residents of Seoul, South Korea. Acta Neurol Scand.

[22] Kankirawatana P (1999). Epilepsy awareness among school teachers in Thailand. Epilepsia.

[23] Lee H, Lee SK, Chung CK, Yun SN, Choi-Kwon S (2010). Familiarity with, knowledge of, and attitudes toward epilepsy among teachers in Korean elementary schools. Epilepsy Behav.

[24] Abulhamail AS, Al-Sulami FE, Alnouri MA, Mahrous NM, Joharji DG, Albogami MM (2014). Primary school teacher's knowledge and attitudes toward children with epilepsy. Seizure.

[25] Aydemir N (2011). Familiarity with, knowledge of, and attitudes toward epilepsy in Turkey. Epilepsy Behav.

[26] Jensen R, Dam M (1992). Public attitudes toward epilepsy in Denmark. Epilepsia.

[27] Caveness WF, Gallup GH (1980). A survey of public attitudes toward epilepsy in 1979 with an indication of trends over the past thirty years. Epilepsia.

[28] Canger R, Cornaggia C (1985). Public attitudes toward epilepsy in Italy: results of a survey and comparison with U.S.A. and West German data. Epilepsia.

[29] Zanni KP, Matsukura TS, Maia Filho HS (2012). Beliefs and Attitudes about Childhood Epilepsy among School Teachers in Two Cities of Southeast Brazil. Epilepsy Res Treat.

[30] Akpan MU, Ikpeme EE, Utuk EO (2013). Teachers' knowledge and attitudes towards seizure disorder: a comparative study of urban and rural school teachers in Akwa Ibom State, Nigeria. Niger J Clin Pract.

[31] Ghanean H, Nojomi M, Jacobsson L (2013). Public awareness and attitudes towards epilepsy in Tehran, Iran. Glob Health Action.

[32] Tiamkao S, Aaauevitchayapat N, Arunpongpaisal S, Chaiyakum A, Jitpimolmard S, Phuttharak W (2005). Knowledge of epilepsy among teachers in Khon Kaen Province, Thailand. J Med Assoc Thai.

[33] Chung K, Ivey SL, Guo W, Chung K, Nguyen C, Nguyen C (2010). Knowledge, attitudes, and practice toward epilepsy (KAPE): a survey of Chinese and Vietnamese adults in the United States. Epilepsy Behav.

